# SIO management algorithm for patients with overweight or obesity: consensus statement of the Italian Society for Obesity (SIO)

**DOI:** 10.1007/s40519-016-0279-3

**Published:** 2016-04-21

**Authors:** Ferruccio Santini, Luca Busetto, Barbara Cresci, Paolo Sbraccia

**Affiliations:** Obesity Center, Endocrinology Unit, University Hospital of Pisa, Pisa, Italy; Department of Medicine, University of Padua, Padua, Italy; Section of Diabetology, Careggi University Hospital, Florence, Italy; Department of Systems Medicine, Medical School, University of Rome Tor Vergata, Via Montpellier 1, 00133 Rome, Italy

In approaching the treatment of obesity, three major caveats, specific to this complex disease, need to be taken into consideration in order to avoid over-simplification.

Firstly, obesity definition is currently based on the body mass index (BMI). However, BMI has two major limitations: it is not a measure of fat mass, and it does not convey any information on fat distribution and regional fat depots. These limitations are well known by the scientific community that is struggling to find ways to move beyond BMI in obesity classification.

Secondly, for the reasons specified above, the development of comorbidities or complications, which occur in the vast majority of obese patients during the course of the disease, is not always linearly correlated with BMI. Many variables contribute to their manifestation beyond the degree of obesity: duration of disease, age, sex, fat distribution, genetic background, the degree of mechanical disability, etc.

Thirdly, treatment options are now quite few. Their indications should take into account the severity of obesity together with the presence and severity of complications and age, in order to grade interventions; these varying from therapeutic lifestyle changes to bariatric surgery.

In order to provide a staging system able to help clinicians in phenotyping obese patients, beyond BMI, Sharma and Kushner [1] developed the so-called EOSS (Edmonton Obesity Staging System) composed of the following five stages:

0. No apparent obesity-related risk factors (e.g., blood pressure, serum lipids, fasting glucose, etc., within normal range), no physical symptoms, no psychopathology, no functional limitations and/or impairment of well-being.
1. Presence of obesity-related subclinical risk factors (e.g., borderline hypertension, impaired fasting glucose, elevated liver enzymes, etc.), mild physical symptoms (e.g., dyspnea on moderate exertion, occasional aches and pains, fatigue, etc.), mild psychopathology, mild functional limitations, and/or mild impairment of well-being.
2. Presence of established obesity-related chronic disease (e.g., hypertension, type 2 diabetes, sleep apnea, osteoarthritis, reflux disease, polycystic ovary syndrome, anxiety disorder, etc.), moderate limitations in activities of daily living and/or well-being.
3. Established end-organ damage such as myocardial infarction, heart failure, diabetic complications, incapacitating osteoarthritis, significant psychopathology, significant functional limitations, and/or impairment of well-being.
4. Severe (potentially end-stage) disabilities from obesity-related chronic diseases, severe disabling psychopathology, severe functional limitations, and/or severe impairment of well-being.

The EOSS has been validated as a system able to identify patients at increased mortality risk who therefore deserve more clinical and therapeutic attention [2].

We have taken advantage of this now well-established staging system to develop a therapeutic algorithmic chart (Fig. 1) that includes BMI, age and EOSS stages. At each intersection, a color code identifies the proposed preferred treatment option. Obviously, treatment options are not mutually exclusive, but have to be understood as additive (e.g., a patient eligible for bariatric surgery should continue to follow therapeutic lifestyle changes and, if needed, pharmacotherapy).

**Fig. 1:**
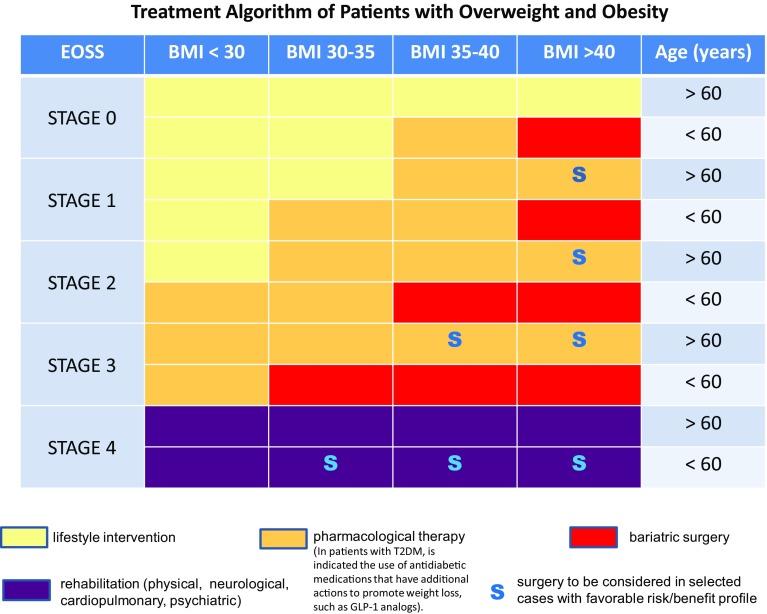
Treatment algorithm chart that takes advantage of the EOSS (Edmonton Obesity Staging System, see text and Ref. [1]). At each intersection a color code identifies the proposed preferred treatment option. Obviously, treatment options are not mutually exclusive but have to be understood as additive

## Strengths and limitations

The strength of the EOSS system relies on its ability to better identify patients who are at increased risk of mortality [2]. The limitations of the EOSS system have been clearly highlighted by Sharma and Kushner in their review paper [1]. They recognize that definitions of some risk factors are subject to change. Furthermore, the EOSS system includes subjective parameters, such as psychological impact or functional performance, the assessment of which may vary among clinicians. In this regard, attention should be drawn to the vagueness of certain definitions such as mild psychopathology, anxiety disorder, significant psychopathology, and severe disabling psychopathology. In addition, the lack of any reference to eating disorders, in particular binge eating disorder, should be pointed out which since 2013 has been considered an autonomous diagnostic category by DSM-5.

By integrating the EOSS system, our therapeutic algorithmic chart includes its pros and cons. In addition, a specific limitation of our chart is the lack of evidence-based data.

## Conclusion

We believe that chronic diseases such as obesity have to be tackled with flexibility and understanding. Any treatment option should be thoroughly explained to the patient, sharing with him the rationale and cost–benefit ratio behind any proposed treatment; treatment that has to be ultimately tailored to the individual patient. However, any algorithm that may help clinicians in their delicate choices is always welcome. We hope this would be the case for our chart.

Finally, we did not indicate any level of evidence or strength of recommendation, since the proposed chart is based on expert opinions and since evidence is at the moment insufficient.
